# Increasing Skeletal Muscle Mass in Mice by Non-Invasive Intramuscular Delivery of Myostatin Inhibitory Peptide by Iontophoresis

**DOI:** 10.3390/ph16030397

**Published:** 2023-03-06

**Authors:** Kohki Michiue, Kentaro Takayama, Atsuhiko Taniguchi, Yoshio Hayashi, Kentaro Kogure

**Affiliations:** 1 Department of Pharmaceutical Health Chemistry, Faculty of Pharmaceutical Sciences, Tokushima University, Shomach-1, Tokushima 770-8505, Japan; 2 Department of Environmental Biochemistry, Kyoto Pharmaceutical University, Yamashina, Kyoto 607-8414, Japan; 3 Department of Medicinal Chemistry, Tokyo University of Pharmacy and Life Sciences, Hachioji, Tokyo 192-0392, Japan; 4 Department of Pharmaceutical Health Chemistry, Graduate School of Biomedical Sciences, Tokushima University, Shomach-1, Tokushima 770-8505, Japan

**Keywords:** sarcopenia, myostatin inhibitory peptides, iontophoresis, intramuscular deliver, peptide delivery, weak electricity, transdermal

## Abstract

Sarcopenia is a major public health issue that affects older adults. Myostatin inhibitory-D-peptide-35 (MID-35) can increase skeletal muscle and is a candidate therapeutic agent, but a non-invasive and accessible technology for the intramuscular delivery of MID-35 is required. Recently, we succeeded in the intradermal delivery of various macromolecules, such as siRNA and antibodies, by iontophoresis (ItP), a non-invasive transdermal drug delivery technology that uses weak electricity. Thus, we expected that ItP could deliver MID-35 non-invasively from the skin surface to skeletal muscle. In the present study, ItP was performed with a fluorescently labeled peptide on mouse hind leg skin. Fluorescent signal was observed in both skin and skeletal muscle. This result suggested that the peptide was effectively delivered to skeletal muscle from skin surface by ItP. Then, the effect of MID-35/ItP on skeletal muscle mass was evaluated. The skeletal muscle mass increased 1.25 times with ItP of MID-35. In addition, the percentage of new and mature muscle fibers tended to increase, and ItP delivery of MID-35 showed a tendency to induce alterations in the levels of mRNA of genes downstream of myostatin. In conclusion, ItP of myostatin inhibitory peptide is a potentially useful strategy for treating sarcopenia.

## 1. Introduction

Sarcopenia is defined as a loss of muscle mass and function, and it is a major public health and economic issue that mainly affects older adults [1,2]. Inducing muscle production is an important strategy in the treatment of sarcopenia. Exercise and dietary supplements are useful ways to induce muscle production [3], but other treatments that regulate muscle growth are necessary to increase muscle mass in patients with sarcopenia.

Myostatin is one possible therapeutic target for increasing muscle growth. Myostatin (growth differentiation factor-8, GDF-8) is a member of the transforming growth factor-β (TGF-β) superfamily and negatively regulates muscle growth by binding to activin type I and type II receptors and thus activating Smad 2/3 signaling [4,5,6,7,8]. Accordingly, myostatin inhibitors, including antibody, recombinant protein, and peptide, have been shown to be useful tools for inducing muscle growth, leading to the promising treatment of muscle atrophic disorders, such as muscular dystrophy, sarcopenia, and cancer-associated cachexia [9,10,11,12,13,14,15,16,17,18,19,20]. However, clinical use for improving muscle wasting have not been achieved yet; hence, there is still room for the new strategies. In our works, several types of myostatin inhibitory peptides have been developed so far [21]. One *retro-inverso* 16-mer peptide, myostatin inhibitory D-peptide-35 (MID-35; lrxkrwirxkiwriyw-amide, x = D-cyclohexylglycine, lowercase letter = D-amino acid), has been shown to exert an especially potent myostatin inhibitory effect and to exhibit high stability against biodegradation [21]. In a mouse model, single injection of a solution of MID-35 into the tibialis anterior muscle led to a weight increase in 1.3-fold after 28 days [21]. Thus, MID-35 is a promising candidate for the treatment of sarcopenia; however, intramuscular injection is an invasive and technically challenging procedure. Thus, a non-invasive and simple intramuscular delivery technology would be suitable for MID-35 to be developed as a sarcopenia therapy.

A transdermal drug delivery system (TDDS) would be suitable for non-invasive intramuscular delivery of MID-35. However, the primary physical barrier for transdermal drug delivery is the skin. The skin is composed of the epidermis and dermis, and the stratum corneum on the surface of the epidermis is the greatest barrier to drug penetration. In general, small molecules with a molecular weight of 500 Dalton or less can penetrate the skin, but transdermal penetration of larger molecules is difficult [22]. Therefore, various TDDS technologies have been developed to enhance drug penetration into the skin [23]. 

There are two types of TDDS technology: passive and active methods. Passive methods consist of penetration-enhancing reagents and nanoparticles. Treatment of the skin with penetration enhancers, such as ethylene vinyl acetate, improves the penetration of the drug molecules. However, skin irritation from the penetration enhances is a drawback of this technique. Various nanoparticles have been developed as drug carriers for TDDS. The nanoparticles for TDDS include liposomes, ethosomes, transfersomes, niosomes, lipid nanoparticles and polymeric nanoparticles. However, encapsulation processes must be tailored to different drugs, and drug release from the nanoparticles must be controlled. 

On the other hand, active TDDS methods include microneedles, electroporation and iontophoresis. Microneedles physically destroy the stratum corneum of the epidermis with numerous needles 150–1500 μm in length. Microneedles are composed of metals, polymers and biodegradable materials, such as hyaluronic acid. Although microneedles are expected to be a potential TDDS technology, there are concerns regarding irritation, infection and limited drug loading. The electroporation method uses high-voltage (50–1500 V) electricity to form transient pores to facilitate the penetration of drug molecules. The skin burns caused by the high-voltage electricity are a problem in clinical application to the human body. Iontophoresis (ItP) is a non-invasive transdermal drug delivery technology that uses a weak electric current (0.3–0.5 mA/cm^2^) [24,25,26]. Electro-repulsion and electro-osmosis are considered as the mechanisms of penetration enhancement by weak electricity. However, prolonged use of ItP may cause skin irritation. Therefore, all TDDS technologies have advantages and disadvantages.

We focused on ItP as a non-invasive method for the potential intramuscular delivery of MID-35. Previously, we have demonstrated the utility of ItP for the delivery of various macromolecules, such as siRNA, liposomes and antibodies [27]. siRNA has been delivered into the skin by ItP and induced significant knocking down of the expression of target interleukin 10 mRNA. This indicates that siRNA was delivered to not only intercellular space, but also to the cytoplasm of the skin cells by ItP. Regarding the mechanism, we found that the weak electric current of ItP induces the activation of cell signaling pathways such as protein kinase c (PKC) through changes in the cell membrane potential and cation influx such as Ca^2+^ via transient receptor potential (TRP) channels. Furthermore, gap junction protein connexin 43 significantly decreased after its phosphorylation and significant filamentous actin depolymerization was induced by weak electric current treatment. It was suggested that macromolecules such as siRNA and liposomes can penetrate into intercellular spaces cleaved by weak electric currents. Therefore, we concluded that ItP would promote transdermal penetration of macromolecules by cleaving intercellular junctions through activation of cell signaling pathways via Ca^2+^ influx [27].

We also found that the weak electric current induces unique endocytosis, such as tubular endocytosis, for the uptake of extraneous macromolecules. In addition, weak electric current treatment of culture cells in vitro and rats in vivo increased ceramide levels [27]. Ceramide is known to form pores in the mitochondrial outer membrane [28]. Based on these findings, it has been speculated that macromolecules such as siRNA with a molecular weight of less than 70,000 can leak from the endosomes induced by weak electric current through the pores composed of ceramide. Previously, we performed the isobaric tags in relative and absolute quantification proteomic analysis for identifying changes in the protein phosphorylation levels occurred during weak electric current mediated cellular uptake. It was found that heat shock protein 90 (Hsp90) α and myristoylated alanine-rich C-kinase substrate (Marcks) were highly phosphorylated after weak electric current treatment. We have also shown that weak electric current induced Rho GTPase activation via Hsp90 and PKC, which could contribute to cellular uptake by actin cytoskeleton remodeling [27]. 

As mentioned above, because antibodies, with an MW of approximately 150,000, were delivered by ItP into deep regions of the skin [27], we expected that MID-35 (MW of approximately 2350) would be non-invasively delivered via ItP to the skeletal muscle from the surface of the skin. In the present study, we examined the intramuscular delivery of a fluorescently labeled peptide by ItP from the skin surface of the hind legs of mice. Furthermore, the effects of the ItP-mediated intramuscular delivery of MID-35 on the mass and fiber distribution of the tibialis anterior muscle of mice were evaluated. Mechanisms leading to the effects of ItP-delivered MID-35 on muscle were evaluated by examining the expression of mRNA from genes associated with muscle production. Taken together, our results suggest that ItP represents a novel intramuscular delivery strategy for medicines, including myostatin inhibitory peptides for the treatment of sarcopenia.

## 2. Results

### 2.1. Delivery of a Peptide to the Skeletal Muscle from the Skin Using ItP

To observe the ability of ItP to deliver peptides to the skeletal muscle, we used an FITC-labeled myostatin inhibitory peptide with the sequence WYIEWIKIQIWSKLRL. We attempted to deliver this peptide non-invasively from the surface of the skin to the skeletal muscle by ItP on the hind legs of C57BL/6J mice.

**Figure 1:**
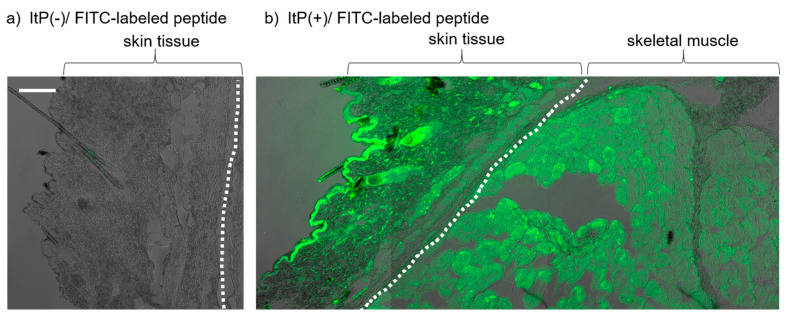
Intradermal and intramuscular distribution of fluorescently labeled myostatin inhibitory peptide after ItP: (**a**) Skin section treated with FITC-labeled peptide topical application without ItP. (**b**) A section containing skin and muscle after ItP of FITC-labeled peptide on the surface of the skin of mouse hind legs. The sections were observed by confocal laser scanning microscope 42 days after application. The white dotted line shows the border between skin and muscle. White bar = 100 μm.

**Figure 2:**
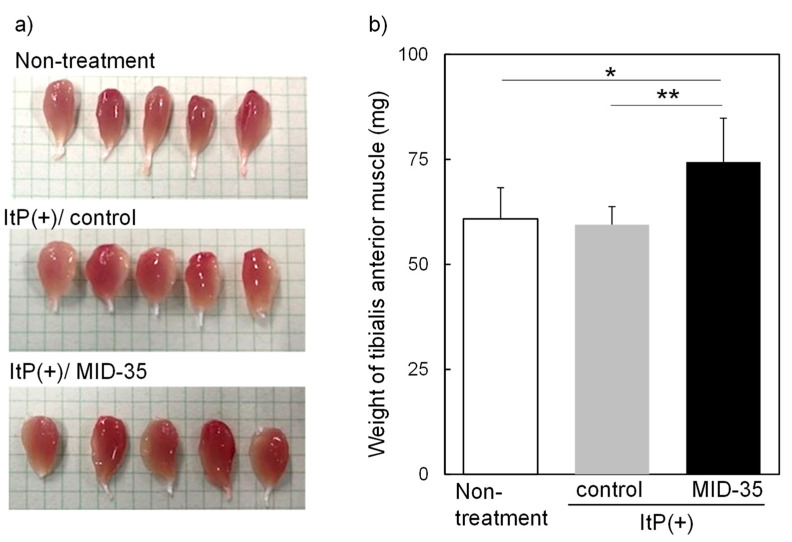
Effect of iontophoretic intramuscular delivery of MID-35 on (**a**) appearance and (**b**) weight of tibialis anterior muscle of mice: The tibialis anterior muscle was collected from mice hind legs on day 42 after performing ItP of MID-35 three times. Then, the weight of the muscle tissue was measured. Quantitative data are presented as mean ± SD from at least three independent experiments. * *p* < 0.05, ** *p* < 0.01.

After performing ItP for 1 h, the skin and muscle tissue under the peptide-containing anode was collected, and frozen tissue sections were observed with a confocal laser scanning microscope. In the case of topical application of FITC-labeled peptide solution on the skin surface, no fluorescence signal was observed in the skin section (Figure 1a). On the other hand, fluorescence of FITC was observed widely in the skin and muscle sections of regions treated by ItP with the FITC-labeled peptide (Figure 1b). The fluorescence was widely distributed and was observed even in deep regions of the muscle, at least 1000 μm from the skin surface. The FITC-labeled peptide solution was placed under the anode as mentioned in the Materials and Methods section due to the fact that the peptide had positive charges (basic amino acids, such as lysin and arginine). However, FITC does not have a positive charge. Even if free FITC was released from the FITC-labeled peptide by degradation, it would not be able to penetrate the skin under anodal electrode by ItP. Therefore, the fluorescent signal observed in Figure 2 indicates that the FITC-labeled peptide was delivered into the skin or muscle.

### 2.2. Effect of ItP of MID-35 on Skeletal Muscle

Given the potential delivery of FITC-labeled peptides to skeletal muscle by ItP, we used ItP to administer MID-35 to the hind legs of mice. MID-35 is a latest peptide consisting entirely of D-amino acids and has a sequence, and similar molecular weight (approximately 2.100 kDa) [21]. Among the myostatin inhibitory peptides we have developed, MID-35 is the most effective and stable peptide. ItP of MID-35 was performed three times, and to examine the effect of this procedure on skeletal muscle, the weights of the gastrocnemius muscle and tibialis anterior muscle were measured on day 42. The weight of the gastrocnemius muscle of mice treated with the peptide increased slightly as compared to that of ItP/control mice, but the difference was not statistically significant (Supplementary Figure S1). On the other hand, the size of appearance of the tibialis anterior muscle after ItP of MID-35 was larger than that from non-treated and ItP/control mice (Figure 2a). In the case of the tibialis anterior muscle, the weight increase following treatment with ItP/MID-35 (1.25-fold) was statistically significant, although ItP/control did not show any effect on the muscle weight (Figure 2b).

**Figure 3:**
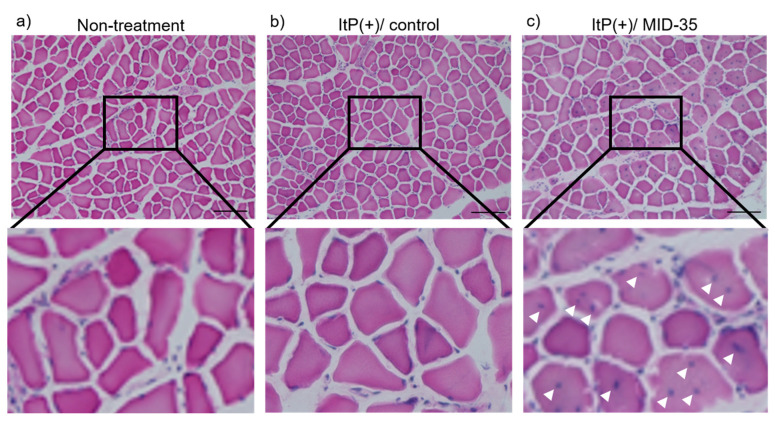
Effect of iontophoretic intramuscular delivery of MID-35 on muscle fiber morphology: Muscle tissues were collected from (**a**) non-treated mice, (**b**) control mice treated with ItP and PBS (ItP(+)/control) and (**c**) mice treated with ItP/MID-35 (ItP(+)/MID-35). The muscle sections were stained with HE and observed by light microscopy. The upper three images were collected at 100× magnification, and the lower three images are magnified view. White arrows indicate the central nuclei. The length of the black bar is 100.

We next evaluated the effect of ItP/MID-35 on the morphology of the tibialis anterior muscle by HE staining. No blue dots indicating central nuclei were observed in the HE-stained cross-sections of tibialis anterior muscle fibers isolated from non-treated or ItP/control mice (Figure 3a,b). On the other hand, tibialis anterior muscle sections from mice treated with ItP/MID-35 exhibited blue central nuclei throughout the HE-stained sections (Figure 3c, white arrows).

**Figure 4:**
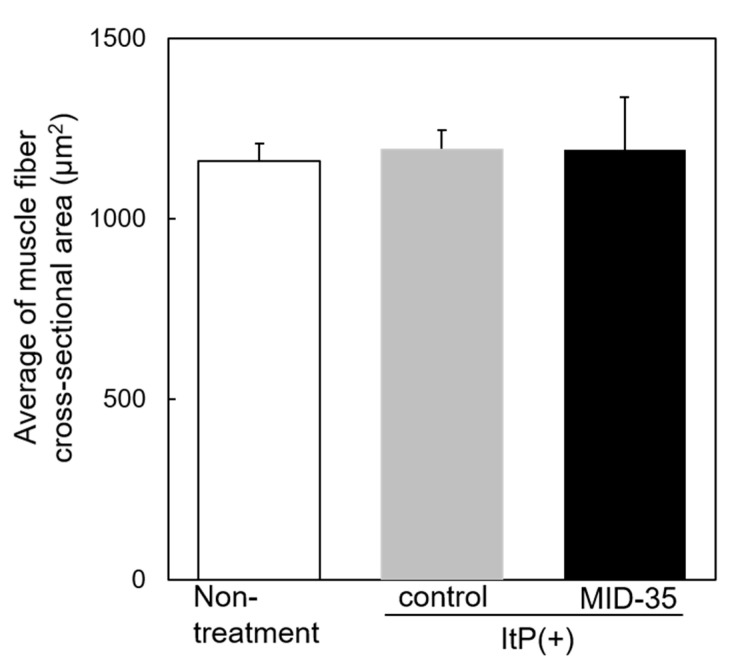
Effect of the delivery of MID-35 by ItP on muscle fiber cross-sectional area: The cross-sectional areas of the muscle fiber shown in Figure 3 were calculated with the image analysis software ImageJ. The columns of white, gray and black are the cross-sectional areas of muscle collected from non-treated mice, control ItP/PBS-treated mice and ItP/MID-35-treated mice, respectively. The values are shown as means ± SD obtained from five different images of muscle cross-sections.

We then evaluated the effect of ItP/MID-35 on the area of muscle fiber cross-sections. The average of the area of the cross-section of muscle fibers from mice treated with ItP/MID-35 was similar to that from non-treated and ItP/control mice (Figure 4).

**Figure 5:**
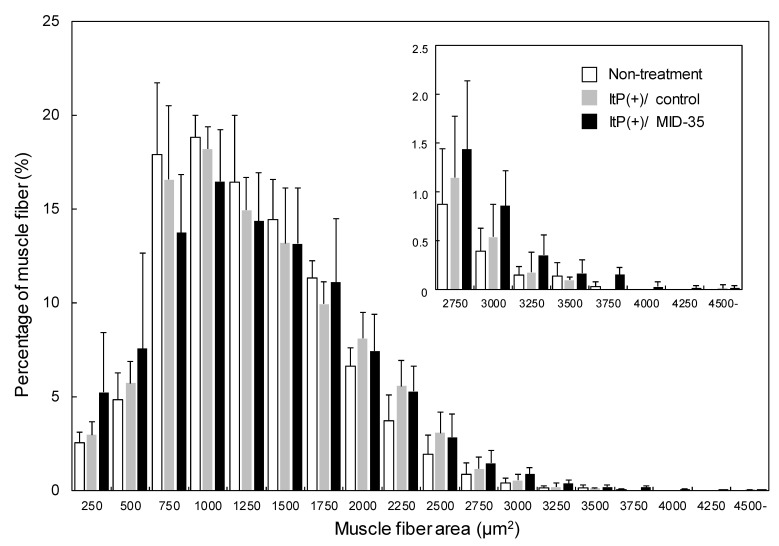
Analysis of the distribution of muscle fiber cross-sectional areas after delivery of MID-35 by ItP: The percentages of muscle fiber having the noted sizes from the analysis in Figure 4 were calculated. The columns of white, gray and black are the cross-sectional areas of muscle collected from non-treated mice (Non-treatment), control ItP/PBS-treated mice (ItP(+)/control) and ItP/MID-35-treated mice (ItP(+)/MID-35), respectively. The values are shown as means ± SD obtained from five different images of muscle cross-sections.

In order to analyze the muscle fibers in more detail, the distribution of cross-section areas of muscle fiber from mice treated with ItP/MID-35 was compared to the distribution from non-treated and ItP/control mice. The percentages of muscle fiber with areas between 250 and 500 μm^2^ and greater than 2750 μm^2^ in mice treated with ItP/MID-35 tended to be higher compared to those from non-treated and ItP/control mice, although the differences were not statistically significant (Figure 5).

### 2.3. Effect of ItP of MID-35 on mRNA Expression of Genes Associated with Muscle Growth and Myostatin Signaling Pathways

**Figure 6:**
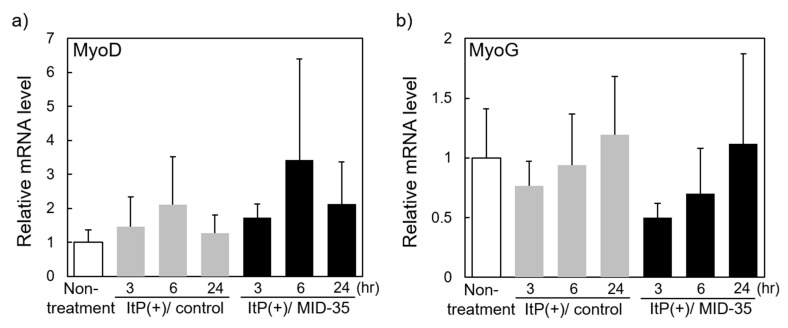

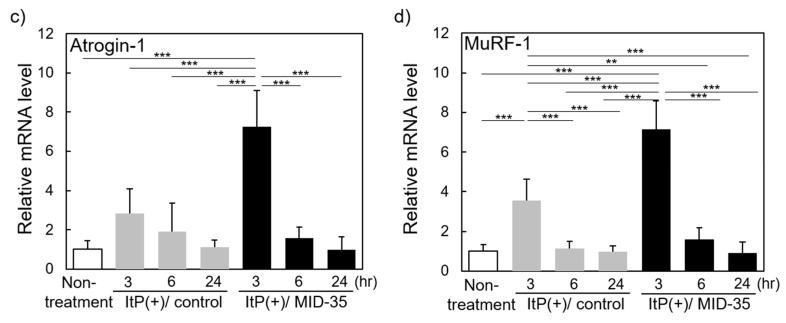
Effect of ItP of MID-35 on the expression of genes related to muscle growth: RNA was extracted from tibialis anterior muscle of the mice at 3, 6 and 24 h after ItP of MID-35. The mRNA levels of (**a**) MyoD, (**b**) MyoG, (**c**) Atrogin-1 and (**d**) MuRF-1 were quantified by real-time RT-PCR. The values are shown as means ± SD from at least three independent experiments. ** *p* < 0.01, *** *p* < 0.001.

We examined the effect of ItP of MID-35 on the expression of genes encoding proteins that regulate muscle growth and proteins in the myostatin signaling pathway. RNA was extracted from the muscle at 3, 6 and 24 h after ItP of MID-35, and mRNA of the muscle differentiation regulator genes MyoD (Myod) and MyoG (Myog) [29,30], and the muscle-specific ubiquitin ligases Atrogin-1 (Fbxo32) and MuRF-1 (Trim63) [31], were quantified by real-time RT-PCR. The expression of both MyoD and MyoG changed over time in the experimental mice, although the changes were not statistically significant. MyoD mRNA showed a tendency to increase transiently at 6 h after ItP, and the change seemed to be slightly greater in the presence of MID-35 (Figure 6a). In contrast to MyoD, MyoG mRNA tended to decrease transiently at 3 h after ItP, and this change also seemed to be somewhat greater in the presence of MID-35 (Figure 6b). On the other hand, the expression of Atrogin-1 and MuRF-1 mRNA increased at 3 h after ItP, but both mRNA levels decreased at 6 h, and the changes in both gene expressions were temporal. MID-35 significantly enhanced the transient changes in Atrogin-1 and MuRF-1 gene expressions (Figure 6c,d). The mRNA levels of Antrogin-1 and MuRF-1 increased unexpectedly by ItP, although Antrogin-1 mRNA change was not statistically significant. Previously, we reported that weak electric current of ItP activates cell signaling pathways [27]. Thus, ItP probably increased transiently expression of various genes including Atrogin-1 and MuRF-1 via activation of cell signaling.

## 3. Discussion

In the present study, we endeavored to increase skeletal muscle mass by the non-invasive intramuscular delivery of the myostatin inhibitory peptide MID-35 by ItP. At first, we examined the effectiveness of ItP using an FITC-labeled myostatin inhibitory peptide (MW: approximately 2100). FITC-labeled peptides that were administered from the surface of the skin became widely distributed throughout not only skin but also muscle (Figure 1b). This result suggests that peptides can be non-invasively delivered to deep regions of skeletal muscle by ItP. This is the first time that ItP has been shown to deliver biomolecules from the skin surface to muscles. Thus, ItP would be a useful technology to non-invasively deliver biological molecules to skeletal muscle from the skin surface. 

Next, we examined the effect of MID-35 delivered by ItP on skeletal muscle. Although the weight of gastrocnemius muscle did not change, the mass of the tibialis anterior muscle was found to have increased significantly (1.25-fold) on day 42 after ItP of MID-35 (Figure 2a,b). This result indicates that MID-35 peptide was delivered to skeletal muscle as the intact form by ItP and the delivered MID-35 functioned in skeletal muscle. In previous reports, the weight of the tibialis anterior muscle was found to increase by a similar amount (1.33-fold) upon intramuscular injection of MID-35 [20], though it should be noted that the dose of MID-35 for ItP (75 nmol) was higher than that for intramuscular injection (30 nmol). Thus, we succeeded in increasing skeletal muscle weight by delivering MID-35 with the non-invasive intramuscular delivery technology ItP in this study.

With regard to the morphology of the skeletal muscle after ItP/MID-35 treatment, many central nuclei were recognized in the muscle fibers (Figure 3c). Central nuclei are known to be an index of muscle fiber production [32]. Although the average muscle fiber area in mice treated with ItP/MID-35 was almost the same as that of non-treated and ItP/control mice (Figure 4), the distribution of kinds of muscle fibers was different. In particular, mice treated with ItP/MID-35 tended to have higher percentages of fine, newly produced muscle fibers (with areas between 250 and 500 μm^2^) and thick, matured muscle fibers (with areas greater than 2750 μm^2^) compared to non-treated and ItP/control mice, although the differences were not statistically significant (Figure 5). These results suggest that treatment with ItP/MID-35 may induce the production and maturation of muscle fibers.

On the other hand, myostatin inhibition is expected to inhibit the transcription of genes related to myostatin. In this study, we measured the effect of ItP/MID-35 treatment on the expression of mRNAs encoding atrogin-1 and muscle-specific RING-finger protein-1, two E3 ubiquitin ligases involved in the limiting of protein growth. As muscle production was induced by ItP/MID-35 treatment (Figure 2), we predicted that the expression of these mRNAs would be inhibited by MID-35. Contrary to our expectation, the expression levels of both genes were temporarily increased at 3 h after ItP, and MID-35 enhanced this temporal activation (Figure 6c,d). These transient responses may represent alterations due to the weak electrical impulses, as well as a compensation for the myostatin inhibition. As the reasons for these complex responses to ItP/MID-35 remain unclear, more detailed investigations will be required in the future.

## 4. Materials and Methods

### 4.1. Materials and Animals

Myostatin inhibitory peptide labeled at the N-terminus with fluorescein isothiocyanate (FITC) and aminocaproic acid and with an amidated C-terminus (FITC-ε -Acp-WYIEWIKIQIWSKLRL-amide) was synthesized by Peptide Institute Inc. (Osaka, Japan). Five-week-old male C57BL/6J mice were purchased from Japan SLC, Inc. (Shizuoka, Japan), and five male mice, the average weight of which was 20 g, were used in each group: non-treated group, ItP/control group and ItP/MID-35 group. 

### 4.2. Synthesis of MID-35

The myostatin inhibitory D-peptide, MID-35, was chemically synthesized by a 9-fluorenylmethoxycarbonyl (Fmoc)-based solid-phase peptide synthesis method as previously reported [21]. Briefly, Fmoc-amino acids were sequentially coupled to Fmoc-NH-SAL resin (Watanabe Chemical Industries, Ltd., Hiroshima, Japan) by using 1-hydroxy benzotriazole (HOBt)-*N*,*N*’-diisopropylcarbodiimide (DIPCI) to prepare the protected peptide-bound resin. The crude MID-35 obtained by trifluoroacetic acid treatment was purified by reverse-phase HPLC. After lyophilization, MID-35 with >95% purity was obtained as a white powder.

### 4.3. Iontophoresis (ItP) Procedure

ItP of peptides was performed according to our previous report [33]. C57BL/6J mice were anesthetized by isoflurane inhalation, and chloral hydrate dissolved in PBS was administered (400 mg/kg mouse) by intraperitoneal injection to complete anesthesia. Hind leg hair was shaved with electric hair clippers. Then, the mouse limbs were fixed in the lateral decubitus position. For ItP of peptides, nonwoven fabric containing 0.1 mg (in 40 μL 10% acetic acid) of FITC-labeled peptide solution was placed as an anode on the shaved leg skin, and nonwoven fabric moistened with 50 μL of PBS was placed on the skin as a cathode. In the case of MID-35, nonwoven fabric containing 0.18 mg (in 50 μL deionized distilled water) of MID-35 solution was placed as an anode on the leg skin. Each nonwoven fabric containing peptide or PBS was attached to Ag-AgCl electrode (3M Health Care, Minneapolis, MN, USA), and the electrodes were connected to a power supply (TCCR-3005, TTI Ellebeau Inc., Tokyo, Japan). ItP was performed with a constant current of 0.34 mA/cm2 for 1 h.

### 4.4. Analysis of Fluorescent Dye Distribution in the Tissue after ItP

The tissue, including skin and muscle, under the anode was collected after ItP of the FITC-labeled peptide and was embedded in OCT compound (Sakura Finetek Japan Co.,Ltd., Tokyo, Japan), followed by freezing with a dry ice–ethanol bath. Then, frozen tissue sections (10 μm) were prepared with a cryostat. After mounting the sections with Dako Fluorescence Mounting Medium (Dako North America, Inc., Carpinteria, CA, USA), FITC fluorescence in the tissue section was observed by confocal laser scanning microscopy (LSM700, Carl Zeiss, Jena, Germany).

### 4.5. Analysis of Muscle Tissue after ItP of MID-35

ItP of MID-35 (75 nmol) was performed three times, on days 0, 7 and 14. The tibialis anterior muscle under the anode was collected on day 42, and the muscle mass was determined. The muscle tissue was soaked in a 30% sucrose solution at 4 °C for 3 h, and frozen muscle sections were prepared as described above. Hematoxylin-eosin (HE) staining of the muscle sections was performed as described previously [33]. Briefly, the muscle sections were stained with Mayer’s hematoxylin solution for 10 min at room temperature, washed with distilled water, and stained with a 1% eosin Y solution for 1 min at room temperature. Then, the muscle sections were dehydrated with 80–100% ethanol, cleared with xylene, and mounted with hydrophobic mounting medium (Entellan New^®^, Merck KGaA, Darmstadt, Germany). The mounted muscle sections were observed with a phase contrast microscope (BZ-9000, Keyence, Osaka, Japan).

### 4.6. Quantitation of mRNA in the Muscle after ItP of MID-35

After collection of the tibialis anterior muscle of the mice treated with peptides by ItP, RNA was extracted as described previously [33]. Briefly, the tibialis anterior muscle was homogenized in QIAzol Lysis reagent using a TissueRuptor II (QIAGEN, Hilden, Germany). After a 5 min incubation at room temperature, total RNA was purified and extracted with the RNeasy Plus Universal Midi Kit. The total RNA concentration was determined using a Nanodrop 8000 spectrophotometer (Thermo Fisher Scientific, Waltham, MA, USA).

Real-time RT-PCR was performed with the extracted RNA by first synthesizing cDNA with PrimeScript RT Master Mix (Perfect Real Time) (Takara Bio, Kyoto, Japan) using an MJ Mini Personal Thermal Cycler (Bio-Rad, Hercules, CA, USA). The reverse transcription reaction was performed at 37 °C for 15 min, and the reverse transcriptase was then inactivated by heating at 85 °C for 5 s. Real-time RT-PCR was performed with TB GreenTM Premix Ex TaqTM II (TliRNaseH Plus) (Takara Bio, Kyoto, Japan) using a Thermal Cycler Dice Real Time System III (Takara Bio). The PCR primers, which are shown in Table 1, were synthesized by Eurofins Genomics. The mRNA levels of MyoD (Myod), MyoG (Myog), Atrogin-1 (Fbxo32) and MuRF-1 (Trim63) were calculated using the 2-ΔΔCt method by normalization relative to GAPDH mRNA levels.

### 4.7. Statistical Analysis

Statistical differences were evaluated by one-way analysis of variance with Tukey’s post hoc test. Statistical significance was calculated from raw data of 5 mice in each group using the software Kaleida Graph ver. 4.5.2 (Synergy Software). Data are presented as mean ± standard deviation (SD). Differences for which *p* < 0.05 were considered statistically significant.

## 5. Conclusions

We demonstrated the ability of ItP to deliver myostatin inhibitory peptide to skeletal muscle. The weight of the skeletal muscle increased 1.25-fold following ItP of MID-35. The proportion of new and mature muscle fibers showed a tendency to increase, and the levels of mRNA of genes downstream of myostatin action tended to be altered by ItP of MID-35. Thus, ItP of myostatin inhibitory peptide may be a useful strategy for treating sarcopenia. Although there are many publications about myostatin inhibitors including peptides, this is the first time that iontophoresis has been shown to non-invasively deliver biomolecules including peptides from the skin surface to the muscles. Therefore, iontophoresis would be a useful technology for non-invasive intramuscular delivery of biological molecules.

## Figures and Tables

**Table 1:** Sequence of PCR primers used in this study.

Gene Name	Forward(5′-3′)	Reverse(5′-3′)
MyoD	AGTGAATGAGGCCTTCGAGA	GCATCTGAGTCGCCACTGTA
MyoG	CCTTGCTCAGCTCCCTCA	TGGGAGTTGCATTCACTGG
Atrogin-1	TAGTAAGGCTGTTGGAGCTGATAG	CTGCACCAGTGTGCATAAGG
MuRF-1	CATCTTCCAGGCTGCGAATC	ACTGGAGCACTCCTGCTTGT
GAPDH	GAGGACCAGGTTGTCTCCTG	ATGTAGGCCATGAGGTCCAC

## Data Availability

Data is contained within the article and Supplementary Material.

## Supplementary Materials

The following supporting information can be downloaded at: https://www.mdpi.com/article/10.3390/ph16030397/s1, Figure S1 Effect of iontophoretic intramuscular delivery of MID-35 on weight of gastrocnemius muscle of mice.
