# Supplementary material for: Increasing Skeletal Muscle Mass in Mice by Non-Invasive Intramuscular Delivery of Myostatin Inhibitory Peptide by Iontophoresis
Source: Pharmaceuticals (Basel). 2023 Mar 6;16(3):397. doi: 10.3390/ph16030397 (PMC10058260; doi:10.3390/ph16030397)
Supplement: Supplementary file 1 [file pharmaceuticals-16-00397-s001.zip › pharmaceuticals-2133425-supplementary.pdf]

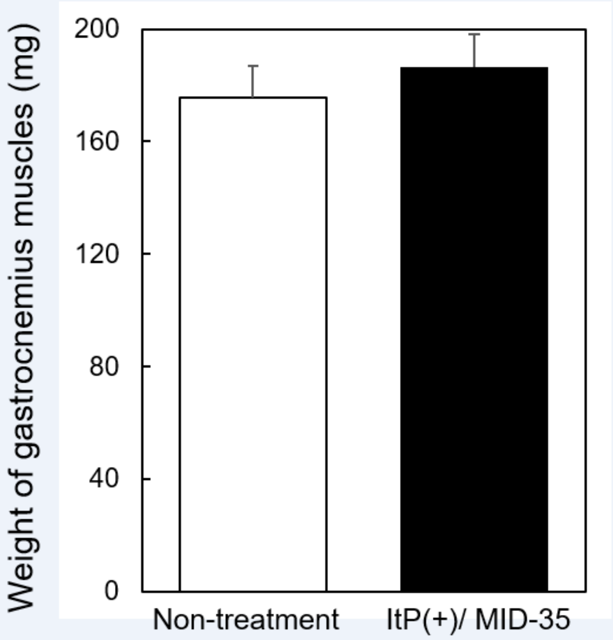

Figure S1. Effect of iontophoretic intramuscular delivery of MID-35 on weight of gastrocnemius muscle of mice: The gastrocnemius muscle was collected from mice hind legs on day 42 after performing ItP of MID-35 three times, then the weight of muscle was measured. The values in the graph are means ± S.D. obtained from at least three different experiments.
